# Outpatient psychotherapy for home-living vulnerable older adults with depression: study protocol of the PSY-CARE trial

**DOI:** 10.1186/s12877-020-01661-1

**Published:** 2020-08-05

**Authors:** Paul Gellert, Ann-Kristin Beyer, Christina Tegeler, Claudia Vathke, Johanna Nordheim, Adelheid Kuhlmey, Eva-Marie Kessler

**Affiliations:** 1 Institute for Medical Sociology and Rehabilitation Science, Charité – Universitätsmedizin Berlin, Charitéplatz 1, 10117 Berlin, Germany; 2 MSB Medical School Berlin, Department of Psychology, Rüdesheimer Str. 50, 14197 Berlin, Germany

**Keywords:** Depression in older adults, Homecare, Psychotherapy, Randomized controlled trial, Pragmatic clinical trial

## Abstract

**Background:**

There is a need to improve psychotherapeutic approaches to treatment for vulnerable older adults with depression in terms of both clinical practice and health care supply. Against this background, PSY-CARE is testing the feasibility and effectiveness of outpatient psychotherapy for home-living older adults in need of care with depression in Berlin, Germany, and neighboring suburban areas.

**Methods:**

In a two-arm single-center pragmatic randomized controlled trial (RCT), manual-guided outpatient psychotherapy will be compared to brief psychosocial counseling. The study population will be compromised of older adults with clinically significant depressive symptoms who have a long-term care grade, as assessed by the German compulsory state nursing care insurance. In the intervention group, individual cognitive-behavioral psychotherapy tailored to the specific needs of this population will be offered by residential psychotherapists as part of the regular healthcare service. In the active control group, participants will receive individual psychosocial telephone counselling and a self-help guide. The planned sample size is *N* = 130 (*n* = 65 participants per group). The reduction of depressive symptoms (primary outcome) as well as the maintaining of activities of daily living, quality of life, and functioning will be assessed with questionnaires provided at baseline, after the end of the intervention and after three months. Feasibility and process evaluation will be conducted qualitatively based on documentation and interviews with psychotherapists, gatekeepers and the participants.

**Discussion:**

PSY-CARE investigates the potentials and limitations of providing outpatient psychotherapeutic treatment meeting the demands of vulnerable home-living older adults with depression under the real conditions of the health care system. The study will provide practical implications to improve access to and quality of outpatient psychotherapy for this poorly supplied population.

**Trial registration:**

The trial is registered at ISRCTN55646265; February 15, 2019.

**Supplementary information:**

**Supplementary information** accompanies this paper at 10.1186/s12877-020-01661-1.

## Background

Depression is a common phenomenon among vulnerable older adults. Compared with the general older population, community-based studies have consistently documented at least two or three times higher rates of depression among older adults with chronic physical conditions, cognitive deficits and/ or sensory limitations, i.e. conditions that often along with functional impairment and dependence on informal and formal support systems for medical care, transport and other essential needs (e.g., [1–5]). Conversely, depression has devastating consequences for older individuals including an increased risk of morbidity and suicide, decreased physical, cognitive and social functioning, all of which are in turn associated with higher rates of service utilization, premature institutionalization and mortality [6, 7].

Meta-analyses and systematic reviews have provided extensive evidence that psychotherapeutic interventions are effective for treating depression in the older population, with strongest evidence for cognitive and behavioral therapy (CBT), life review therapy (LR) and problem-solving therapy (PST) [8, 9]. However, vulnerable older adults with depression including very old, frail and care-dependent older adults were mostly excluded from these studies [10], despite advances for psychotherapeutic research in the context of specific comorbid conditions including chronic obstructive pulmonary disease (COPD), heart failure, Parkinson’s disease, stroke, early stages of dementia and suicidal ideations [for an overview, see [11]]. As a result, psychotherapy for this rapidly growing population is understudied in terms of clinical practice, feasibility, efficiency and effectiveness.

The described deficiencies in research are also mirrored in the massive shortage of mental health care supply for vulnerable older adults with depression [e.g., [12]]. Under the current conditions of health care systems, referrals to outpatient psychotherapy very seldom succeed due to health care providers’ pessimistic attitudes towards vulnerable older patients’ treatability, patients’ inaccessibility, shortage of psychotherapists qualified to work with older adults, and costs (e.g., [13–15]). For example, although outpatient psychotherapy is integral part of public and private health care in Germany, ambulatory psychotherapy is de facto non-existing or only in very exceptional cases among vulnerable older people in need of care [16]. The fact that vulnerable older adults’ mental health care needs are obviously largely unmet is even more problematic as most of them have positive attitudes towards psychotherapy or even prefer psychotherapy to pharmacotherapy, contrary to what is often believed [17, 18].

The significant correlation between depression and impaired quality of life together with increasing numbers of vulnerable older adults and the very poor supply reality highlight the importance of developing and rigorously evaluating appropriate psychotherapeutic approaches, including outpatient psychotherapy for home-living vulnerable older adults.

When it comes to clinical practice, existing studies on mental health services for homebound older adults and nursing-home participants has clearly shown the necessity of gatekeepers (such as aging services networks for patients to gain access to mental health care providers), interprofessional collaboration, caregiver involvement and in-home treatment (for patients who cannot freely leave their home [19, 20]). Furthermore, vulnerable older patients have been shown to profit from CBT and PST through encouragement of adaptive coping skills together with behavioral activation and increased exposure to pleasant events (e.g., [21–23]). Finally, research on LR in long-term care has shown the importance of reminiscence and biographical interventions for patients in order to identify coping skills in their biography as well as to find hope and purpose in life (e.g., [23, 24]).

### Objectives

PSY-CARE investigates the feasibility and effectiveness of individual outpatient psychotherapy within the regular German health care system for home-living older adults in need of care with clinically significant depression. Outpatient psychotherapy tailored to the specific needs of this population is provided based on a treatment manual by certified resident CBT psychotherapists with extensive professional experience in geriatric or geropsychiatric settings.

In terms of the effectiveness of the intervention, we hypothesized that psychotherapy leads to a greater reduction of depressive symptoms, which we considered the primary outcome, compared to an alternative intervention, which encompassed brief psychosocial counselling (Hypothesis 1). Further, we hypothesized that psychotherapy will be associated with a relative difference at follow-up in the secondary outcomes – i.e., quality of life, activities of daily living, functioning and subjective health – in the intervention condition compared with the control condition (Hypothesis 2). More specifically, we assume maintenance of activities of daily living and quality of life, and functioning/ subjective health in the intervention condition relative to the control condition, which we assume shows a decline. Further, in this trial treatment response (50% or greater reduction in depressive symptoms from baseline to follow up) and remission [defined as number of individuals who scoring below the cut-off of 10 points at the Geriatric Depression Scale (GDS; [25, 26] at follow up] will be used as secondary outcomes.

In terms of the feasibility of the intervention under the real conditions of the German health care system, a wide range of approaches to recruit participants of this hard-to-reach population is used and effective versus non-effective recruitment methods are documented. In addition, we aim to explore possibilities and constraints of psychotherapists’ clinical practice of working with home-living vulnerable older adults including their professional experiences with home treatment in case of participants’ limited mobility, as well as collaboration with other healthcare professionals, especially with general practitioners and formal or informal caregivers.

## Methods/design

### Trial design

**Fig. 1:**
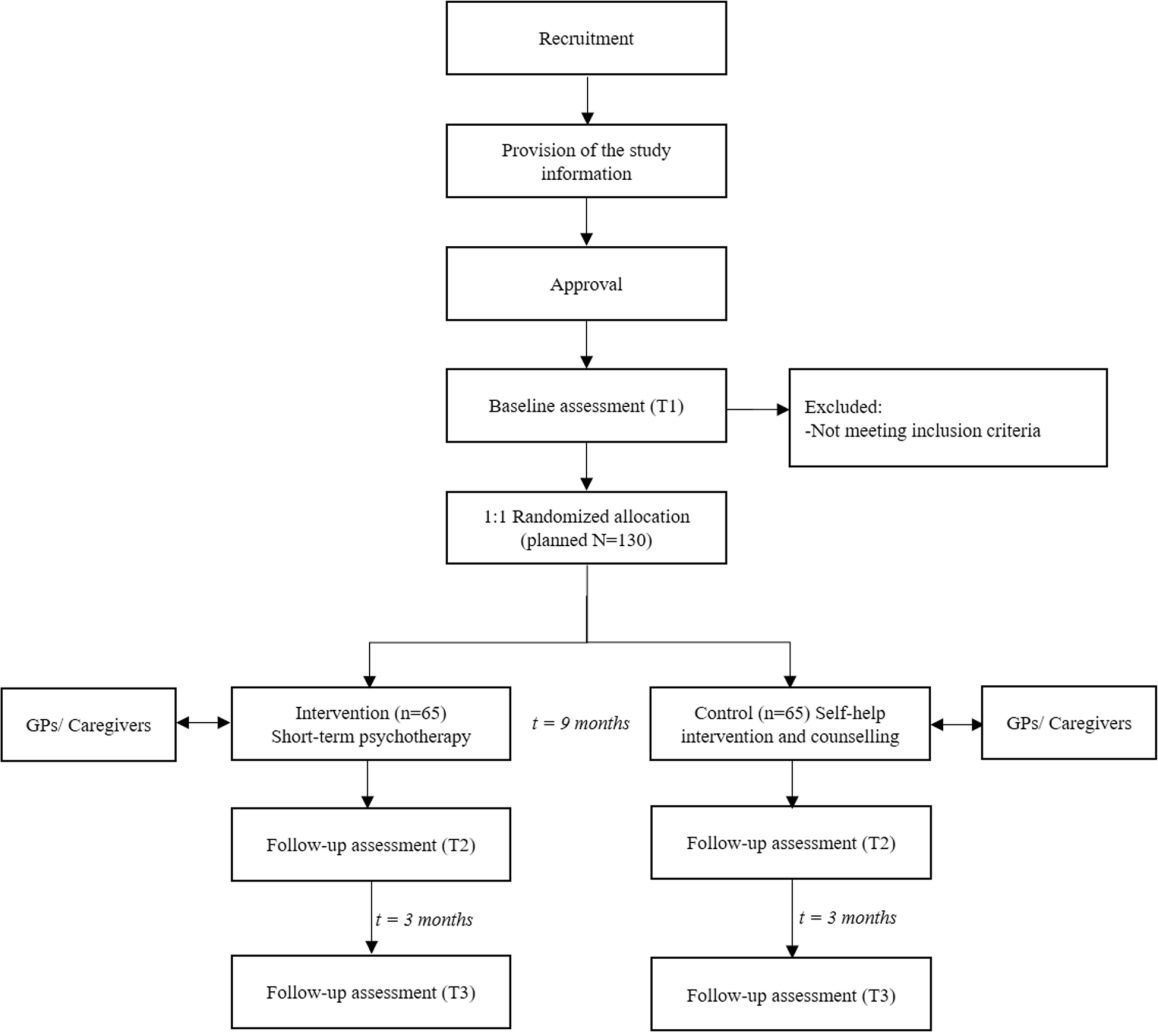
Flow chart of participants of the PSY-CARE trial

PSY-CARE is a single-center interventional study, using a pragmatic randomized controlled design with nested qualitative research, comparing individual psychotherapy with an active control condition. The intervention condition will include manual-guided psychotherapy provided by gerontologically trained resident psychotherapists [27]. The active control condition will include individual telephone counselling carried out by project staff members as well as a self-help guide for participants and their caregivers [28]. The flow chart of the study design is depicted in Fig. 1. The schedule of enrolment, interventions, and assessments is shown in Table 1.

**Table 1:**
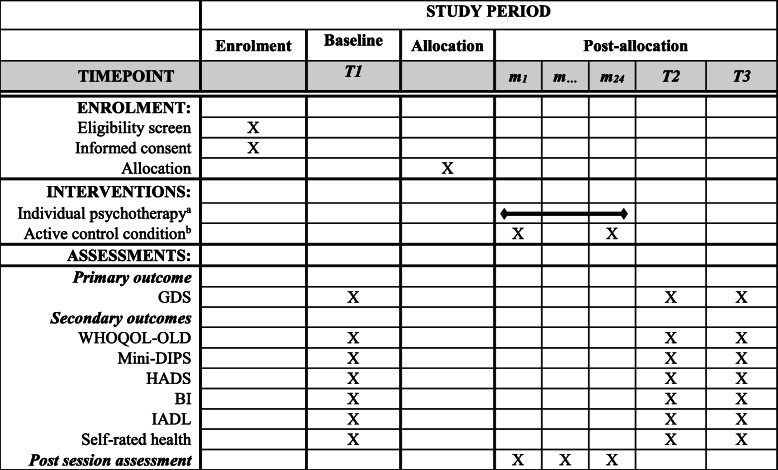
Schedule of enrolment, interventions, and assessments of the PSY-CARE trial

^a^ Three probationary sessions and up to 24 therapy sessions

^b^ Individual telephone counselling, which is offered two times per participant and per caregiver carried out by trained staff members as well as self-help literature for participants and their caregivers

*GDS* Geriatric Depression Scale, *WHOQOL-OLD* single item of the World Health Organization Quality of Life in OLD age. *Mini-DIPS* German structured clinical interview for the diagnose of depressive symptoms. *HADS* Hospital Anxiety and Depression Scale. *BI* Barthel-Index (Activities of daily living). *IADL* Instrumental Activities of Daily Living-Scale at. *T1* Time point 1, baseline. *T2* Time point 2, after the intervention

PSY-CARE is going to be located in Berlin, Germany, and neighboring suburban areas. The study center will be located at the Department of Psychology, MSB Medical School Berlin. The method center (i.e., external evaluation) will be located at the Institute for Medical Sociology and Rehabilitation Science, Charité – Universitätsmedizin Berlin.

### Participants, interventions, and outcomes

#### Study setting

The psychotherapy intervention will take place in psychotherapy practices or – in case of a participant’s impaired mobility or confinement to bed – at participants’ homes (i.e., in that case psychotherapists will be traveling to participants’ homes). The control group will receive treatment via telephone (psychosocial counseling) and postal service (self-help literature; audiobook or printed version).

#### Eligibility criteria

Participants will be included in the study if they [1] are 60 years and older (women/ men) [2]; are living at home (Berlin, Germany) [3]; have a long-term care grade, as assessed by the German compulsory state nursing care insurance (1 = little impairment to 5 = hardship case; see [29]) [4]; show clinically relevant depressive symptoms (major depression; adjustment disorder with depressive symptoms; dysthymia); and [5] are willing to participate in one of the two treatments. Exclusion criteria will be moderate to severe dementia as well as delirium, acute psychosis or other cognitive disorders, mania or hypomania or terminal stage of a disease. Further reasons for exclusion will be currently receiving psychotherapy, communication difficulties and visual or hearing impairments that would severely affect an individual’s capability to take part in the study. Reasons for participant exclusion will be recorded.

#### Interventions

Participants included in the study will be randomly assigned to one of two treatment groups (intervention condition versus active control condition). the intervention condition, outpatient short-term psychotherapy tailored to the specific needs of vulnerable older adults, will be offered by residential CBT psychotherapists with extensive geriatric and geropsychiatric experience as part of the regular healthcare service. After the taking of an anamnesis, a medical examination by a general practitioner or a medical specialist and test sessions, participants will be treated in up to 24 therapy sessions. Psychotherapeutic treatment, as described in the PSY-CARE manual, follows the regular CBT rationale, but is augmented by psychotherapeutic techniques of a) reminiscence, b) empowerment and c) inclusion of participants’ social systems, based on existing effective psychotherapeutic approaches for vulnerable older adults with depression (see above). Before participating in PSY-CARE, psychotherapists will be trained in the assessment of depression in vulnerable older adults and the application of the PSY-CARE Manual.

The active control condition will include individual telephone counselling carried out by project staff members as well as self-help literature for participants and their caregivers. The telephone counselling will be offered two times per participant and per caregiver by members of the project team (all trained certified psychotherapists). In addition, control group participants and their caregivers will receive a print or audio version of a self-help guide based on existing literature on depression in later life [e.g., [30]], which will include psychoeducation and self-help instructions. For participants in the control condition, regular psychotherapy will not be retained if desired.

Critical events for intervention or control participants will be recorded. In order to improve and monitor the adherence to intervention protocol, psychotherapists will be regularly supervised by PSY-CARE supervisors. The project team will stay in contact with participants via telephone or letters on a regular basis.

#### Outcomes

The primary outcome variable is the reduction of depressive symptoms of the participants assessed by the 12-item Geriatric Depression Scale (GDS [31, 32];) at baseline (T1), directly after the intervention (T2), and at follow-up after 3 months (T3) compared to baseline. Total scores range from 0 to 12, a score of 4 or more indicates the presence of clinically relevant depressive symptoms.

Secondary outcome variables are participants’ [1] quality of life measured by a single item from the World Health Organization Quality of Life in old age, i.e. WHOQOL-OLD [2, 33]; the diagnose of depressive symptoms (major depression; adjustment disorder with depressive symptoms; dysthymia; F32, F33, F34, F43), assessed by a German structured clinical interview for psychological diseases (Mini-DIPS [34];) as well as depressive symptoms (and comorbid anxiety) assessed with the Hospital Anxiety and Depression Scale (HADS [35];) [3] Activities of daily living and functioning measured using the Barthel-Index and the IADL-Scale [36, 37]; and [4] self-rated health measured by a single item asking “How would you estimate your health status in the last 4 weeks?” on a 6-point Likert scale from 1 = “very poor” to 6 = “excellent”. Treatment response (50% or greater reduction in depressive symptoms from baseline to follow up) and remission (defined as number of individuals who scoring below the cut-off of 10 points at the GDS at follow up) will be used as secondary outcomes. All secondary outcomes will be assessed at Baseline (T1), after the intervention (T2), as well as at follow-up after 3 months (T3).

#### Sample size

For sample size estimation, a difference in depression (i.e., primary outcome) between intervention and control group at follow-up of medium effect size is considered as clinically relevant (Cohen’s d = 0.5), which also corresponds to finding from a recent meta-analysis [8]. The sample estimation (test family: t-test for independent samples) with Gpower 3.1 [38] yielded an estimated minimum sample size of the two groups of *n* = 51 at a Type I error rate of alpha = 0.05 and a statistical power of 1-beta = 0.80 individuals. If a dropout rate of 20% is taken into account [39], as was the case in comparable studies, the final rounded sample size per group is *n* = 65, or *N* = 130 persons in total. While 20% of drop out is common in psychotherapy trails for older adults, considering the vulnerability of our sample of vulnerable participants, we will be collecting further participants to guarantee sufficient statistical power at follow up.

#### Recruitment of psychotherapists

The recruitment of resident psychotherapists who volunteer to participate in PSY-CARE is supported by the biggest Association for Behavior Therapy in Germany (German Society for Behavioral Therapy) and includes several offers in newsletters, online mailing lists and distribution of flyers. Certified CBT psychotherapists with extensive work experience in geriatric and/or geropsychiatric settings are included into the pool of PSY-CARE psychotherapists. In addition, they receive a two days training course, conducted by the principal investigator (PI) [40]. They will receive an expense allowance of €1000 for their participation in the study, in addition to their regular fees for psychotherapeutic services within the regular health care system.

#### Recruitment of participants and the treatment team

Recruitment takes place via self-referral as well as gatekeeper referral and uses a wide range of approaches including distributions of flyers and posters, public talks, presentations within institutions, advertisements in online newsletters of collaborating institutions in the health and care sector and media reports in newspapers and magazines. In addition, professionals working with vulnerable home-living older adults on a day-to-day basis in ambulatory care services, medical practices, geriatric and geropsychiatric clinics and counseling centers were recruited via phone call, e-mail or mail. Gatekeepers are given flyers and reminder letters to prompt referrals. In order to participate in the study, an informed consent sheet is signed and participants receive a copy of the signed documents. Neither participants nor gatekeepers will receive any direct financial compensation. The main caregiver of the participant (professional caregiver or family member) is included in the study, if the participants give their written consent for participation.

### Assignment of interventions

#### Allocation and blinding

Participants will be randomized to the two treatment arms. A computer-assisted randomization to either intervention or control condition with 1:1 ratio is performed by the method center, using R v3.2.1 [41, 42] and the package “blockrand” (block randomization with block sizes 4, 6, and 8). Stratifications is not undertaken. In order to ensure allocation concealment, central randomization is used at the participant level, where the recruiting person got sequentially numbered, opaque, sealed envelopes from the method center. The creation of the random assignment sequence, the enrollment participants and the assignment of the participants to the interventions will be strictly separated. The project staff involved in evaluating the results will be blinded to the intervention allocation.

### Data collection, management and statistical analysis

Standardized assessments will be carried out at baseline, directly after the intervention and at a follow-up after 3 months. The quantitative assessment (Hypotheses 1 and 2) among participants will be carried out by two trained study nurses (clinical psychologists with advanced clinical training). The questionnaires will be conducted as computer assessed interviews using a secure laptop and will take up to a maximum of two hours per participant. Data collection will be monitored by the method center and later automatically transferred to SPSS v25 (IBM, Cary, Ind.). When selecting the measuring instruments, attention was paid to the objectivity, reliability, and validity of the assessments.

Psychotherapists will also complete questionnaires in paper-pencil format and later transferred to a SPSS v25 data mask. Missing data will be handled using multiple imputation. Furthermore, qualitative data will be collected from psychotherapists, gatekeepers and older participants. Data will be stored and analyzed at the method center. Only the evaluation team will have access to the data, as defined in the data protection concept as part of the ethics approval.

The evaluation based on participant data will be carried out by means of multivariate longitudinal methods (random effects models). Both intention-to-treat analyzes and completers analyzes will be carried out. A post hoc effectiveness analysis of three potentially moderating characteristics of those in need of care will be considered: (a) degree of cognitive impairment, (b) drug use, and (c) late-onset versus early onset depression. In addition to effect sizes, remission and response rates (Reliable Change Index; RCI) will be determined.

Alongside the effectiveness trial, qualitative data gathered over recruitment as well as treatment processes will be analyzed in order to provide a systematic analysis of the therapeutic, structural and organizational possibilities and barriers regarding the realization of outpatient psychotherapy within the context of interprofessional cooperation under real-life conditions of the German health care system. Specifically, documentation of approaches to access vulnerable older adults will be compared to one another in order to identify successful versus non-successful approaches. In terms of practical implications, qualitative methods will be used in order to analyze interview and focus group data of psychotherapists’ experiences of providing psychotherapy for their patients. In addition, qualitative methods are used to analyze how patients experience the psychotherapeutic process and its effectiveness. Quantitative and qualitative results will finally be combined (method triangulation).

### Trial status

Recruitment/ enrolment began 25/02/2019 (i.e., first participant in). Currently, we are estimating that the assessment phase will continue until September 2021 (i.e., last participant out) when the last psychotherapy session and T3 assessment has been completed.

### Ethics and dissemination

#### Research ethics approval

This research was approved by the Ethics Committee of the Medical School Hamburg (25/10/2018; MSB-2018/20).

#### Protocol amendments

Important protocol modifications will be reported in the trial paper.

#### Consent or assent

PSY-CARE participants and psychotherapists will receive comprehensive information material on the research project and the trial. Written informed consent to take part in the study will be obtained from participants and certified psychotherapists prior to data collection.

#### Declaration of interests

The authors declare that they have no competing interests. The funder had no role in the design of this study and will not have any role during its execution, analyses, interpretation of the data, or decision to submit results.

#### Access to data

The project team will be given access to the cleaned data set. The datasets generated during and analyzed during the current study will be stored in a non-publically available repository. The access information is available from the PI and the corresponding author on reasonable request. To ensure confidentiality, data dispersed to project team members will be blinded of any identifying participant information.

#### Dissemination policy

Publications are planned in a high-impact peer-reviewed journal.

## Discussion

PSY-CARE is a large international study using a pragmatic randomized controlled design for testing the feasibility and effectiveness of outpatient psychological therapy for home-living vulnerable older adults with clinically significant depression. Going beyond extant psychotherapy studies, a systematic analysis of the therapeutic, structural and organizational possibilities and barriers regarding the realization of outpatient psychotherapy under real-life conditions of the German healthcare system is provided which may in part be transferable to other countries. Overall, the present trial aims to provide real-world evidence on the provision of psychotherapeutic treatment for home-living vulnerable older adults, with the potential of providing practical implications to improve access to and quality of outpatient psychotherapy.

## Supplementary information

**Additional file 1.** PSY-CARE trial: Update to the study protocol.

## Data Availability

The datasets generated during and analyzed during the current study will be stored in a non-publically available repository. The access information is available from the PI and the corresponding author on reasonable request. To ensure confidentiality, data dispersed to project team members will be blinded of any identifying participant information.

## Abbreviations

CBT: Cognitive and behavioral therapy
COPD: Chronic obstructive pulmonary disease
DFG: German Research Foundation
G-BA: Gemeinsamer Bundesausschuss [Fund of the German Federal Joint Committee]
GDS: Geriatric Depression Scale
HADS: Hospital Anxiety and Depression Scale
ID: Identity number
ISRCTN: International Standard Randomised Controlled Trial Number
LR: Life review therapy
Mini-DIPS: Diagnostisches Kurzinterview bei psychischen Störungen [Diagnostic brief Interview for Psychological Diseases]
MSB: Medical School Berlin
PST: Problem-solving therapy
RCI: Reliable change index
RCT: Randomized controlled trial
PSY-CARE: Psychological counselling and therapy for treating depression in homebound older adults
WHOQOL-OLD: World Health Organization Quality of Life in OLD age
