# Supplementary material for: Outpatient psychotherapy for home-living vulnerable older adults with depression: study protocol of the PSY-CARE trial
Source: BMC Geriatr. 2020 Aug 5;20:271. doi: 10.1186/s12877-020-01661-1 (PMC7409457; doi:10.1186/s12877-020-01661-1)
Supplement: Supplementary file 1 — Additional file 1. PSY-CARE trial: Update to the study protocol. [file 12877_2020_1661_MOESM1_ESM.pdf]

PSY-CARE trial: Update to the study protocol

Paul Gellert[1], Christina Tegeler[2], Eva-Marie Kessler[2]

[1]Charité – Universitätsmedizin Berlin, Institute of Medical Sociology and Rehabilitation Science, Germany
[2]MSB Medical School Berlin, Department of Psychology, Germany
Contact: eva-marie.kessler@medicalschool-berlin

Trial registration: The trial is registered at ISRCTN55646265; February 15, 2019 [1]
Date of the original version of the study protocol (published online): August 05, 2020 [2]
Date of the update to the study protocol (written): December 04, 2020

This is an update to the pre-registered [1] study protocol "Outpatient psychotherapy for home-living vulnerable older adults with depression: Study protocol of the PSY-CARE trial" [2]. In this two-arm single-center pragmatic randomized controlled trial (RCT), older adults with clinically relevant symptoms of depression and a long-term care grade have been randomized to either intervention (i.e., individual cognitive-behavioral psychotherapy tailored to the specific needs) or active control (i.e., individual psychosocial telephone counselling and a self-help guide) [2]. Since the middle of the first quarter of 2020, the development of the COVID-19 pandemic has been associated with major challenges for the implementation of the project. Our participants all belong to a central risk group due to their high level of vulnerability (i.e., depression, old age, need of care and likely to be multimorbid). This required structural and content adjustments to the planned study protocol [2] at short notice. The additions and changes to the study protocol have been listed below.

Intervention conditions

In the active control condition, in addition to the two planned contacts, we provided a third contact 2-4 days after the assessment via telephone since July 30, 2019. This phone call was made to buffer some of the insecurities related with the COVID-19 situation and aimed to motivate enrolled participants of the control condition to further participate in the study. In the intervention condition, starting from March 2020, we fostered the switch to telehealth treatment (video/ telephone) in case that face-to-face psychotherapy with protective measures could not be realized with an individual patient (e.g., therapist or patient with non-specific flu symptoms; patients with massive fear of Covid-19 infection).

Due tophysical, social and psychological challenges in the life of the participants, treatment integrity cannot be fully ensured both in the intervention as well as in the control condition. For example, it has often occurred that psychotherapists had to respond immediately to cases of acute crisis.

Assessment and data collection

Due to the COVID-19 measures, since March 16 we changed face-to-face assessments to telephone assessment (T1, T2, T3). This also included that the assessment of therapy goals between participants and psychotherapists was done via telephone. In addition to the planned assessments, we added items with regard to worries about COVID-19 infection (adapted from [4]). In order to capture the refection on the COVID-19 threat, qualitative interviews of the participants on their experience and their coping with the COVID-19 pandemic were conducted. Finally, a brief 12-month follow up (T4) will be implemented, which will focus on the primary outcome (Short-form of the Geriatric Depression Scale [5]) as well as questions regarding health and healthcare status.

Exclusion criteria

Regarding exclusion criteria, the clarification of dementia by telephone before time 1 (T1) was slightly adjusted since June 16, 2020. Since not all Mini-Mental-State-Examination (MMSE) items can be carried out via telephone, in those individuals with unclear MMSE values, we added the Six Item Cognitive Impairment Test (6-CIT [3]).

Recruitment and sample size

In the context of the first lockdown (since March, 13), we had to stop the recruitment two weeks earlier than originally planned. From March 16, no new admissions have been made; however, those participants who were already enrolled were kept in the trial. A total of N = 197 participants were included in the study and randomly assigned to the control or intervention condition. This sample size in post-hoc power analyzes (assumptions:

effect d = .50; alpha = .05 one-sided; test family: t-test for independent samples) resulted in a power of 1-beta = .97 at the baseline. This does not yet take into account a dropout in the course of the study. Taking into account a dropout rate of 20%, as was the case in the a priori power analysis, a power of 1-beta = .80 would still be guaranteed. In addition, statistical measures for dealing with dropouts in the course of the study (i.e., multiple imputation) will be applied in order to maximize the informative value of the data.

Updated ethical approval

We submitted an amendment to the local ethics committee of the Medical School Berlin/ Medical School Hamburg on April 27, 2020, which covered the abovementioned corona-related changes. The amendment was approved on June 2, 2020 (Ref.: MSB-2020/33).

References

[1] PSY-CARE: Psychological counselling and therapy for treating depression in homebound older adults. ISRCTN Trial Registration, doi: 10.1186/ISRCTN55646265

[2] Gellert, P., Beyer, A.-K., Tegeler, C., Vathke, C., Nordheim, J., Kuhlmey, A., & Kessler, E.-M. (2020). Outpatient psychotherapy for home-living vulnerable older adults with depression: Study protocol of the PSY-CARE trial. BMC Geriatrics, 20:271. doi: 10.1186/s12877-020-01661-1

[3] Jefferies, K., & Gale, T. M. (2013). 6-CIT: six-item cognitive impairment test. In Cognitive Screening Instruments (pp. 209-218). Springer, London.

[4] Kessler, E. M., Bowen, C. E., Baer, M., Froelich, L., & Wahl, H. W. (2012). Dementia worry: a psychological examination of an unexplored phenomenon. Eur J Ageing, 9(4), 275-284. doi: 10.1007/s10433-012-0242-8

[5] Sutcliffe, C., Cordingley, L., Burns, A., Mozley, C. G., Bagley, H., Huxley, P., Challis, D. (2000). A new version of the geriatric depression scale for nursing and residential home populations: the geriatric depression scale (residential) (GDS-12R).
